# IL17-Producing γδ T Cells May Enhance Humoral Immunity during Pulmonary *Pseudomonas aeruginosa* Infection in Mice

**DOI:** 10.3389/fcimb.2016.00170

**Published:** 2016-12-06

**Authors:** Tingting Pan, Ruoming Tan, Meiling Li, Zhaojun Liu, Xiaoli Wang, Lijun Tian, Jialin Liu, Hongping Qu

**Affiliations:** Department of Critical Care Medicine, Ruijin Hospital, Shanghai Jiaotong University School of MedicineShanghai, China

**Keywords:** IL17-γδ T cells, B cells, B cell activating factor, immunoglobulin(s)

## Abstract

The host acquired immune response, especially the humoral immunity, plays key roles in preventing bacterial pneumonia in the lung. Our previous research demonstrated that interleukin 17-producing γδ T cells (IL17-γδ T cells) have a protective effect on the early innate immune response during acute pulmonary *Pseudomonas aeruginosa* infection. However, whether IL17-γδ T cells also play a role in humoral immunity is unknown. In this study, an acute pulmonary *P. aeruginosa* infection model was established in wild-type and γδ TCR^−/−^ C57BL/6 mice. The expression of IL-17 on γδ T cells isolated from infected lung tissues increased rapidly and peaked at day 7 after acute infection with *P. aeruginosa*. Compared with wild-type infected mice, the levels of total immunoglobulins including IgA, IgG, and IgM in the serum and BALF were significantly decreased in γδ TCR^−/−^ mice, with the exception of IgM in the BALF. Moreover, CD69 expression in B cells from the lungs and spleen and the level of BAFF in the plasma were also decreased in γδ TCR^−/−^ mice. IL17-γδ T cell transfusion significantly improved the production of immunoglobulins, B cell activation and BAFF levels in γδ TCR^−/−^ mice compared with γδ TCR^−/−^ mice without transfusion; this effect was blocked when cells were pretreated with an IL-17 antibody. Together, these data demonstrate that IL17-γδ T cells are involved in CD19^+^ B cell activation and the production of immunoglobulins during acute pulmonary *P. aeruginosa* infection. Thus, we conclude that IL17-γδ T cells may facilitate the elimination of bacteria and improve survival through not only innate immunity but also humoral immunity.

## Introduction

*Pseudomonas aeruginosa* is a Gram-negative opportunistic pathogen that causes various life-threatening infections in critical care units, especially pneumonia patients (Hong et al., 2015). *P. aeruginosa* is intrinsically resistant to several antimicrobial agents and has the capacity to acquire further resistance mechanisms (Ramakrishnan et al., 2014). The frequency of multidrug-resistant and pan-drug resistant strains of *P. aeruginosa* is high in ICUs and increases mortality, morbidity, and hospital costs (Hong et al., 2015). It has been a great challenge to develop effective drugs to treat pneumonia caused by *P. aeruginosa*, as treatment options are often limited for patients who are immunocompromised or have defective physical barriers (Williams et al., 2010). The most susceptible individuals to *P. aeruginosa* pneumonia include transplant recipients, neutropenic patients undergoing chemotherapy and HIV patients, often suffer from (Duraisingham et al., 2014; Savoia, 2014; Smith et al., 2014). Therefore, immunotherapy has become potent and promising adjunct to standard antimicrobial therapy against infectious diseases.

γδ T cells preferentially localize to epithelial and mucosal tissues and recognize antigens via an MHC unrestricted mechanism (Prinz et al., 2013). Through their induction of cytokines and chemokines, γδ T cells promote the differentiation and activation of monocytes, neutrophils and dendritic cells, which are involved in pathogen clearance. Depletion of γδ T cells leads to impaired host defense to lung infections by *Klebsiella pneumonia* (Moore et al., 2000), *Staphylococcus aureus* (Cheng et al., 2012) and *Mycobacterium tuberculosis* (Lockhart et al., 2006). Our previous studies found that interleukin 17-producing γδ T cells (IL17-γδ T cells) promoted neutrophil chemotaxis to enhance innate immunity and eliminate bacteria during acute *P. aeruginosa* infection in mice (Liu et al., 2011, 2013).

However, clearance of *P. aeruginosa* from the respiratory system requires both innate and adaptive immunity (Jensen et al., 2010). Patients with acquired immune deficiency, such as HIV patients, are more susceptible to *P. aeruginosa* infections (Movahedi et al., 2016). HIV patients with *P. aeruginosa* pneumonia are also more likely to become bacteraemic. In the adaptive immune response, humoral immunity is believed to protect the respiratory system from microbial infection and systematic dissemination via production of specific antibodies against the pathogen (Akcay et al., 2009). In addition to the neutralization of the pathogens, specific antibodies facilitate the removal of pathogens by phagocytes and activate the complement pathway to kill the pathogens (Ricklin et al., 2010). Approximately 20% of antibody deficient patients have had *P. aeruginosa* infections (Duraisingham et al., 2014), and it has also been reported that patients with selective IgA deficiency have a high risk of disseminated pseudomonal infections (Williams et al., 2010; Duraisingham et al., 2015).

Previous studies have shown that the levels of some immunoglobulins increase remarkably when γδ T cells were co-cultured with B cells (Brandes et al., 2003). It has also been reported that γδ T cells induce expression of essential B cell co-stimulatory molecules (Caccamo et al., 2006). An interesting study found that TCRα^−/−^ mice still efficiently develop germinal centers and produce immunoglobulins (Wen et al., 1994). These studies suggest that γδ T cells play import roles in humoral immunity by enhancing the activity of specific antibody-producing B cells. However, the role of γδ T cells, especially IL17-γδ T cells in humoral immunity during acute *P. aeruginosa* infection is unknown.

In this study, we built an acute *P. aeruginosa* lung infection model in γδ TCR knockout (γδ TCR^−/−^) and wild-type mice, and investigated the effect of adoptive transfer of IL17-γδ T cells isolated from wild-type mice. We verified the role of IL17-γδ T cells in humoral immunity and investigated if this role specifically required IL-17. In recent years, numerous preclinical and clinical trials of γδ T cell immunotherapy have been performed in various malignancies. Under these setting, γδ T cell immunotherapy may become a potent and promising adjunct to standard antimicrobial therapy against acute *P. aeruginosa* infection.

## Materials and methods

### Materials and animals

Frozen aliquots of PAO1 (*P. aeruginosa* strain 1, a derivative of the original Australian PAO isolate, provided by Y.Q. Xu, Shanghai Jiao Tong University, China) were used for all intranasal inoculations. Pathogen-free C57BL/6 mice were purchased from the Animal Laboratory Center, Shanghai Institutes for Biological Sciences, Chinese Academy of Sciences (Shanghai, PR China) and C57BL/6 γδ TCR^−/−^ mice (Stock Number: 002120) were purchased from the Jackson Laboratory (Farmington, CT, USA). The mice were verified to have complete loss of T cells bearing TCR γδ chains in prior study (Itohara et al., 1993). All animal procedures were approved by the University Committee for Laboratory Animals in accordance with the guidelines of the Shanghai Institutes for Biological Sciences Council on Animal Care. The animals were separated into four groups: Wild type (WT) group, γδ TCR^−/−^ (KO) group, γδ TCR^−/−^ transfused with IL17-γδ T cells (KO+T) group, γδ TCR^−/−^ transfused with IL17-γδ T cells pretreated with an anti-IL17 antibody (KO+T+A) group.

### Mouse model of pneumonia

Mice were anesthetized by an intraperitoneal injection of a freshly prepared solution of ketamine hydrochloride and xylazine, and were intranasally infected with *P. aeruginosa*. While the mouse was held in an upright position, 10 μl of a bacterial suspension of 10^8^ CFU ml^−1^ was placed in each nostril (total of 20 μl per mouse). In some experiments, anti-murine IL-17 antibody (1 μg/μl, clone 50104; R&D Systems, Minneapolis, MN, USA) was injected intraperitoneally 24 h before and 72 h after the infection in order to neutralize IL-17 in *vivo*. Bronchoalveolar lavage fluid (BALF) and serum were prepared at desired time points as described below. For survival studies, 10 μl of bacterial suspension at 10^10^ CFU ml^−1^ was placed in each nostril of all groups (total of 20 μl per mouse) with 18–20 mice per group. The animals were kept for 4 days after infection and mortality was recorded every 8 h.

### Isolation and adoptive transfer of IL17- γδ T cells

Spleens were removed from healthy C57BL/6 mice, cut into small chunks, grounded gently and filtered through a 70 μm nylon sieve. The resulting single cell suspension was centrifuged at 200 × g for 5 min at 4°C and resuspended in RPMI 1640 medium (Invitrogen, Carlsbad, CA, USA). γδ T cells were further isolated using a γδ T cell-specific cell isolation kit (Miltenyi Biotec, Cologne, Germany) according to the manufacturer's instructions. The purity of the cells was confirmed to be greater than 95% by flow cytometry.

Purified γδ T cells were cultured under conditions of 37°C and 5% CO_2_ in RPMI-1640 medium supplemented with 10% fetal bovine serum (Hyclone Laboratories, Logan, UT, USA), together with a combination of recombinant mouse IL-1β (10 ng/ml) and IL23 (10 ng/ml; both from Peprotech, Rocky Hill, NJ, USA) and anti-TCR γδ (GL-4.5 mg/ml; BD Pharmingen, San Diego, CA, USA). After 3 days, IL17-γδ T cells were first stained with R-phycoerythrin (PE) according to the manufacturer's instructions using the mouse IL-17 Secretion Assay–Detection Kit (Miltenyi Biotec), and then the cells were magnetically labeled with anti-PE microbeads (Miltenyi Biotec) and isolated using a magnetic activated cell sorting separator. Mice were anesthetized by an intraperitoneal injection of a freshly prepared solution of ketamine hydrochloride and xylazine. The purified IL17-γδ T cells (2.5^*^10^6^/ml, total of 20 μl, 5 × 10^4^ cells per mouse) were intranasally transferred to mice as required 24 h prior to infection.

### Flow cytometry

Single-cell suspensions were recovered from lungs as described previously (Liu et al., 2011), followed by red blood cell lysis with an NH_4_Cl/Tris solution. The cells were then fixed with 4% paraformaldehyde, washed twice and stained for surface markers using PE-labeled anti-CD69 (clone H1.2F3) and PE-cy7-labeled anti-CD19 (clone 6D5). Isotype-matched irrelevant antibodies (all from BD Biosciences, San Diego, CA, USA) were used for control staining. The stained cells were analyzed using a FACSCalibur flow cytometer (BD Biosciences) with CellQuest software.

### Quantification of serum and BALF proteins

To quantify serum and BALF, mice were euthanized at desired times post infection. Carefully inserted the needle (a 23–25 gauge needle and a 1 ml syringe) into the posterior vena cava and drew blood slowly until the vessel wall collapsed. After pausing to allow the vein to refill, blood drawing was repeated three or four times or until no more blood was available. Serum was separated and stored at −80°C for later use. BALF was obtained following cannulation of the trachea and three infusions of 1 ml cold PBS containing 0.5 mM EDTA. During the acquisition of BALF, the operation is slow and gentle to avoid damage to the surrounding blood vessels causing contamination with blood antibodies. A portion of the BALF (100 μl) was plated on LB agar for bacterial colony counts. The remaining BALF was immediately centrifuged, and the supernatant was stored at −80°C for later use. The levels of B cell activating factor of the TNF family (BAFF), IgA, IgG, and IgM in the BALF and serum were measured using specific ELISA kits (R&D Systems, Minneapolis, MN, USA) according to the manufacturer's instructions.

### Quantitation of bacterial load in BALF

A portion of the BALF (100 μl) was plated on LB agar for bacterial colony counts after overnight incubation at 37°C. The lower limit of detection was 1 CFU in 100 μl of the BALF, which corresponded to 10 CFU ml^−1^.

### Statistical analysis

All statistical analyses were performed using GRAPHPAD PRISM 4.00 for Windows (GraphPad Software, San Diego, CA, USA). Multi-group comparisons was all performed using an ANOVA followed by Tukey's multiple comparison test. Survival analysis was performed using the log-rank test, and the survival rate was calculated by the Kaplan–Meier method. A *p*-value < 0.05 was considered statistically significant.

## Results

### The expression of IL-17 on γδ T cells in the lungs significantly increased after *P. aeruginosa* pulmonary infection in wild-type mice

**Figure 1:**
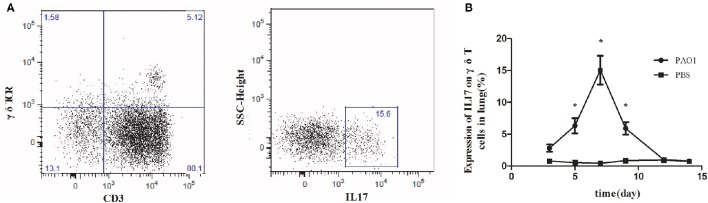
**Increased IL-17 expression on γδ T cells in the lungs in response to ***Pseudomonas aeruginosa*** infection**. C57BL/6 mice were inoculated with 20 μl PAO1 (1 × 10^8^ CFU ml^−1^) or PBS (*n* = 5). At 3, 5, 7, 9, 12, and 14 days following infection, lung mononuclear leukocytes were harvested from the lung, pooled and stimulated with ionomycin and phorbol 12-myristate 13-acetate for 6 h. Lung mononuclear cells were stained with antibodies recognizing CD3, γδ T-cell receptor (γδ TCR), and intracellular IL-17. The cells were first gated on CD3 and γδ TCR (left, **A**) and then the CD3^+^ γδ TCR^+^ cells were gated on IL-17 (right, **A**). The expression of IL1-7 on γδ T cells was measured **(B)**. ^*^p-value < 0.05. These results represent three independent experiments.

We investigated the expression of IL-17 on γδ T cells in the lungs of wild-type C57BL/6 mice during the immune response (day 0–14) against the non-lethal *P. aeroginosa* pulmonary challenge (Liu et al., 2011). The expression of IL-17 on γδ T cells isolated from infected lung tissues increased rapidly and peaked at day 7 after acute infection with *P. aeruginosa* (Figure 1).

### IL17-γδ T cells lead to increased production of IgA, IgG, and IgM in the serum and BALF after *P. aeruginosa* pulmonary infection

**Figure 2:**
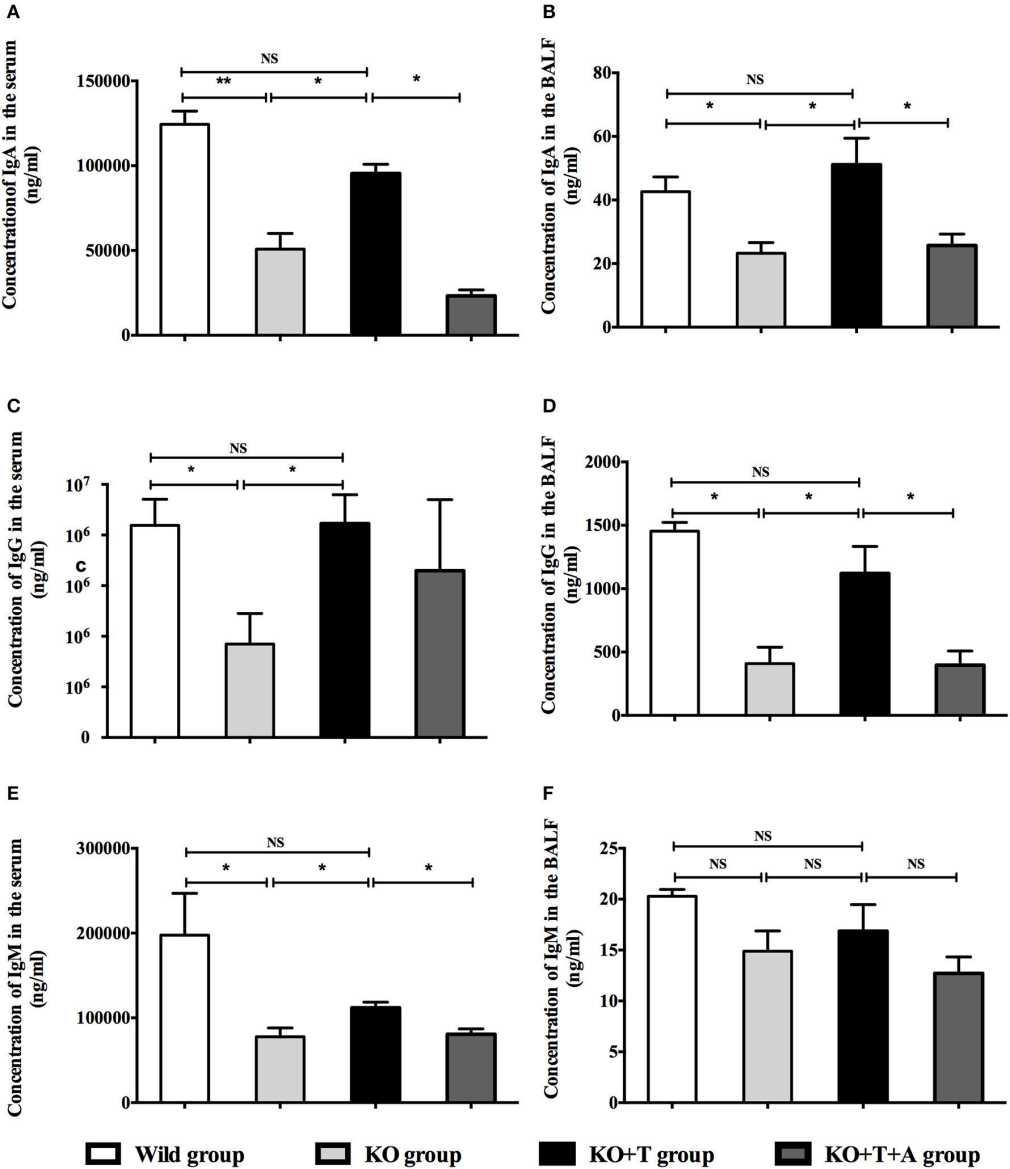
**Effect of IL17-γδ T cells on IgA, IgG, and IgM production**. The mice were divided into four groups: Wild type (WT) group, γδ TCR^−/−^ (KO) group, γδ TCR^−/−^ transfused with IL17-γδ T cells (KO+T) group, γδ TCR^−/−^ transfused with IL17-γδ T cells pretreated with an anti-IL17 antibody (KO+T+A) group. All mice were inoculated with 20 μl PAO1 (1 × 10^8^ CFU ml^−1^). The mice in the KO+T group and the KO+T+A group were transfused with IL17-producing γδ T cells (5 × 10^5^ cells per mouse) 24 h before infection. Anti-murine IL-17 antibody (100 mg) was first administered 24 h before infection and again 72 g after infection in the KO+T+A group. On day 7 post infection, the level of IgA, IgG, and IgM in the serum and BALF of all groups were measured using ELISA. **(A,B)** Levels of IgA in the serum and BALF. **(C,D)** Levels of IgG in the serum and BALF. **(E,F)** Levels of IgM in the serum and BALF. ^*^p < 0.05, ^**^p < 0.01. These results represent three independent experiments.

To investigate the roles of IL17-γδ T cells in humoral immunity, the animals were separated into four groups: Wild type (WT) group, γδ TCR^−/−^ (KO) group, γδ TCR^−/−^ transfused with IL17-γδ T cells (KO+T) group, γδ TCR^−/−^ transfused with IL17-γδ T cells pretreated with an anti-IL17 antibody (KO+T+A) group. We established the acute *P. aeruginosa* pulmonary infection model, and then measured the levels of IgA, IgG, and IgM in the serum and BALF on day 7 post infection. The concentrations of IgA, IgG and IgM in the serum and BALF were significantly higher in the WT group than the KO group, with the exception of IgM in the BALF (Figure 2). The reduced levels of IgA, IgG, and IgM in the KO group were restored by the transfusion of IL17-γδ T cells (Figure 2). However, when pretreated with IL-17 antibody, the effect of IL17-γδ T cell transfusion was blocked except with regards to serum IgG levels (Figure 2). Therefore, after infection with *P. aeruginosa*, IL17-γδ T cells are required for increased production of total IgA, IgG, and IgM in the peripheral blood and lungs, with the exception of IgM in the BALF.

### IL17-γδ T cells influence the activation of B cells and BAFF production during *P. aeruginosa* pulmonary infection

**Figure 3:**
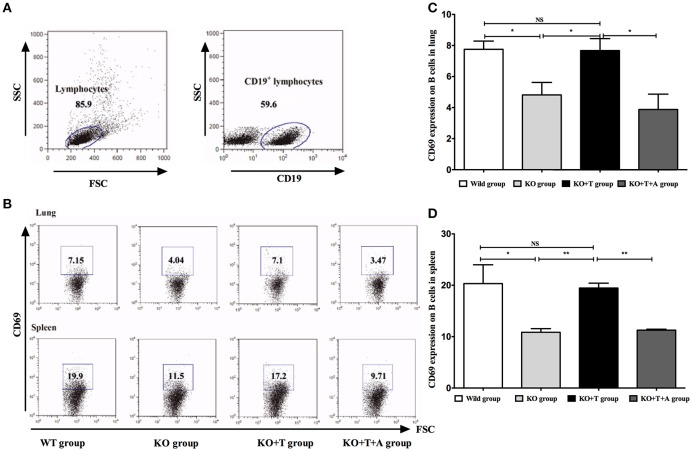
**Effect of IL17-γδ T cells on B cells activation**. The mice in all groups were processed as described in Figure 2. On day 7 post infection, CD69 expression in CD19+ B cells from the lung and spleen were measured. **(A,B)** Representative dot plots of CD69 staining in CD19+ B cells. **(C)** Percentage of CD19+ B cells in the lung expressing CD69. **(D)** Percentage of CD19+ B cells in the spleen expressing CD69. ^*^p < 0.05, ^**^p < 0.01. These results represent three independent experiments.

CD69 is a marker of B cell activation (Krug et al., 2007), and we measured its expression in CD19^+^ B cells to explore the involvement of IL17-γδ T cells in B cell activation during acute *P. aeruginosa* infection. Compared with the WT group, the levels of CD69 in CD19^+^ B cells in the lung and spleen on day 7 post infection were significantly decreased in the KO group (WT vs. KO: Lung, *p* < 0.05; spleen, *p* < 0.05, Figure 3). When IL17-γδ T cells were transfused, B cell activation in the KO+T group was rescued (KO vs. KO+T: Lung, *p* < 0.05; spleen, *p* < 0.01, Figure 3). However, the effect of IL17-γδ T cell transfusion was blocked if the mice were pretreated with IL-17 antibody (KO+T vs. KO+T+A: Lung, *p* < 0.05; spleen, *p* < 0.01, Figure 3).

**Figure 4:**
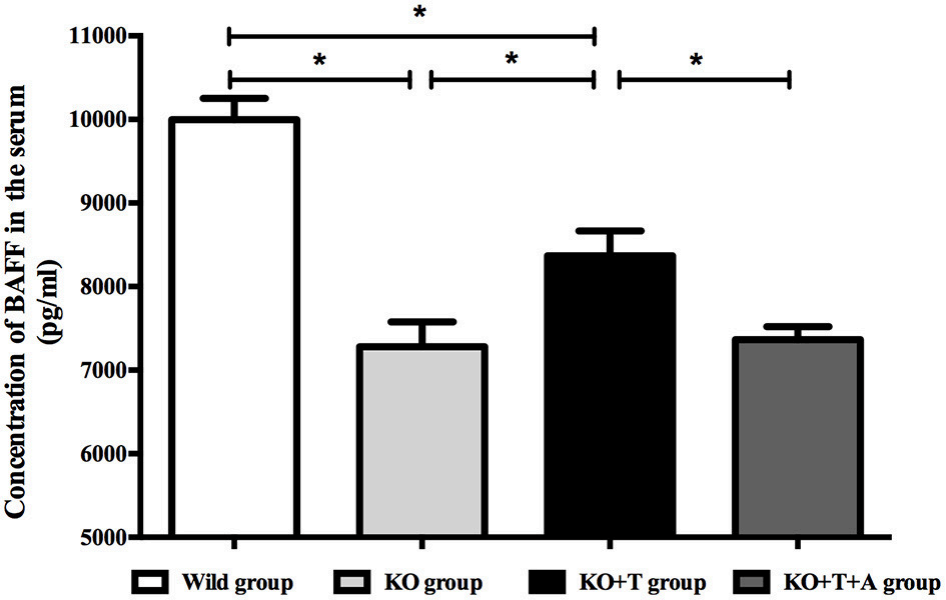
**Effect of IL17-γδ T cells on BAFF production**. The mice in all groups were processed as described in Figure 2. Serum BAFF levels on day 7 post infection were measured using ELISA. ^*^p < 0.05. These results represent three independent experiments.

BAFF, which is best known as a crucial cytokine for B cell activation and maturation (Ng et al., 2004), was measured in the serum of infected mice on day 7 post infection. Consistent with previous results, serum BAFF concentrations were lower in the KO group than the WT group (WT vs. KO, *p* < 0.05, Figure 4). BAFF concentrations in the KO+T group were partially restored by the transfusion of IL17-γδ T cells (KO vs. KO+T, *p* < 0.05, Figure 4). This effect was lost when mice were pretreated with IL-17 antibody (KO+T vs. KO+T+A, *p* < 0.05, Figure 4). Together, these data suggest that IL17-γδ T cells participate in B cell humoral immunity by influencing B cell activation during pulmonary *P. aeruginosa* infection.

### IL17-γδ T cells contribute to the clearance of *P. aeruginosa* after pulmonary infection

**Figure 5:**
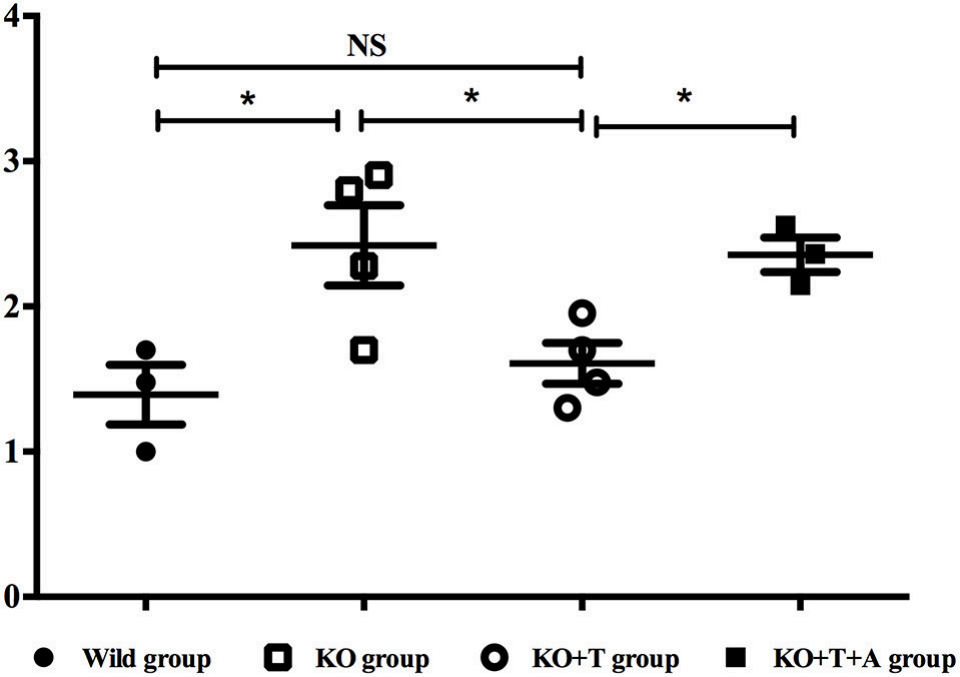
**Effect of IL17-γδ T cells on bacterial clearance**. BALF was obtained on day 7 post infection following cannulation of the trachea and three infusions of 1 ml cold PBS containing 0.5 mM EDTA. A portion of the BALF (100 μl), which corresponded to 10 CFU ml^−1^, was plated on LB agar and incubated overnight at 37°C to determine the bacterial load. ^*^p < 0.05. These results represent three independent experiments.

To assess the role of IL17-γδ T cells in the clearance of *P. aeruginosa*, we measured bacterial loads in pulmonary tissue from the infected mice on day 7 post infection. Compared with the WT group, the KO group presented with significantly increased bacterial loads (WT vs. KO, *p* < 0.05, Figure 5). After being transferred with IL17-γδ T cells, bacterial loads in the KO+T group were restored to levels comparable to the WT group. The bacterial load in the KO group was 8-fold higher than the bacterial load in KO+T group (KO vs. KO+T, *p* < 0.05, Figure 5). However, when the animals were pretreated with IL-17 antibody, the effect of IL17-γδ T cell transfer was lost (KO+T vs. KO+T+A, *p* < 0.05, Figure 5).

### IL17-γδ T cells improve the survival of mice with pulmonary *P. aeruginosa* infection

**Figure 6:**
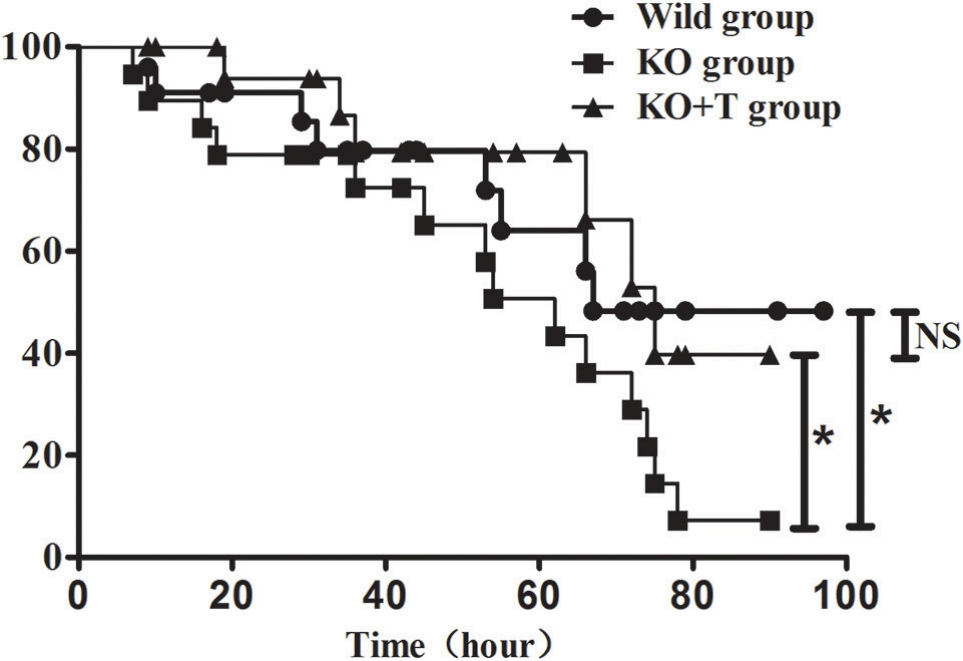
**Effect of IL17-γδ T cells on the survival rate of mice with pulmonary ***P. aeruginosa*** infection**. Mice in the four groups were inoculated with 20 μl PAO1 (1 × 10^10^ CFU ml^−1^). Eighteen to twenty mice in each group were observed at least every 8 h until the fourth day post infection. ^*^p < 0.05. These results represent three independent experiments.

We further investigated whether IL17-γδ T cells could improve the 96-h survival rate in a lethal P. aeroginosa pulmonary challenge model. Compared to the WT group, the survival rate was significantly lower in the KO group (WT vs. KO, *p* < 0.05, Figure 6). Moreover, when IL17-γδ T cells were transfused, the survival rate was rescued to that of the WT group (KO vs. KO+T, *p* < 0.05; WT vs. KO+T, *p* > 0.05, Figure 6).

## Discussion

In this study, we addressed a crucial role of IL17-γδ T cells in the humoral immune response to an acute pulmonary *P. aeruginosa* infection in mice. We reported that the increased IL17-γδ T cells present in the lung following infection were involved in CD19+ B cell activation and the production of immunoglobulins including IgA, IgG, and IgM. We also found that the effect of IL17-γδ T cells on humoral immunity was dependent on IL-17. Additionally, it was demonstrated that regulation of IL17-γδ T cells might be a potential immunotherapy target for acute infectious diseases.

The inflammatory cytokine IL-17 plays a critical role in the immune response to infection (Iwakura et al., 2011). Although early studies believed that CD4+ T cells are the primary source of IL-17, it has been subsequently found that γδ T cells are a more potent source of IL-17 (Korn and Petermann, 2012). IL17-γδ T cells function as part of the defense against bacterial infection during innate immunity (Sutton et al., 2012). In a previous study, we clarified the role of IL17-γδ T cells in innate immunity during acute *P. aeruginosa* pulmonary infection, measuring the proportion of IL17-γδ T cells in lung mononuclear leukocytes at 4, 8, and 12 h post infection and demonstrating that IL17-γδ T cells participate in neutrophil chemotaxis to enhance innate immunity (Liu et al., 2011, 2013). To better understand the role of IL17-γδ T cells in acquired immunity, we extended the detection time to 3, 5, 7, 9, 12, and 14 days post infection. We found that the expression of IL-17 on γδ T cells isolated from infected lung increased rapidly, with a peak at day 7 after acute infection with *P. aeruginosa*. The results showed the possibility of IL17-γδ T cell involvement in humoral immunity during acute pulmonary *P. aeruginosa* infection. The role of γδ T cells in humoral immunity has gradually been recognized in recent studies. Human Vδ2^+^γδ T cells can help B cell maturation and antibody production *in vitro*, suggesting they can promote humoral immunity (Bansal et al., 2012). Additionally, IL-17, which is mainly produced by γδ T cells, was found to recruit B cells in mice infected with *P. aeruginosa* (Fleige et al., 2014). Thus, γδ T cells can have great influence not only on innate immunity but also on humoral immunity in response to infectious diseases.

Humoral immunity is the principal specific immune response to defend host against extracellular pathogenic bacteria (Stead et al., 2002). Patients with IgA deficiency may be at risk for disseminated pseudomonal infections (Duraisingham et al., 2015). In our study, IL17-γδ T cells promoted immunoglobulin production, including increased expression of IgA, IgG, and IgM on day 7 post-infection. It has also been shown that the levels of some immunoglobulins increased remarkably when γδ T cells were co-cultured with B cells *in vitro* (Caccamo et al., 2006). γδ T cells can express remarkable levels of co-stimulatory molecules after stimulation, including CD40L, OX40, ICOS, and CD70, whose essential function in humoral immunity are well-documented (Brandes et al., 2003). In this study, γδ T cells may facilitate B cell activation through direct cell-to-cell contact (Brandes et al., 2003).

In addition to this cell-to-cell contact mechanism, we found that IL17-γδ T cells also induce BAFF production, which may also promote B cell activation. BAFF is a crucial cytokine for B cell activation and survival that is secreted by T cells, monocytes, dendritic cells, and neutrophils and exists in either a membrane-bound or secreted form (Ng et al., 2004). In our study, we found that on day 7 post infection B cell activation in the lung and spleen and serum BAFF levels were significantly decreased in γδ TCR^−/−^ mice but could be rescued partially by IL17-γδ T cell transfusion. However, the effect of IL17-γδ T cell transfusion was blocked if the mice were pretreated with IL-17 antibody. Therefore, IL17-γδ T cells influenced BAFF production to induce the activation of B cells during humoral immunity in acute pulmonary *P. aeruginosa* infection. It has been reported that a strong positive correlation exists between serum IL-17 and BAFF levels in systemic lupus erythematous (Vincent et al., 2013). Doreau et al. reported that IL-17 acts synergistically with BAFF to influence B cell differentiation into immunoglobulin-secreting cells (Doreau et al., 2009). Therefore, IL17-γδ T cells may influence humoral immunity by providing cytokines, including BAFF. In future studies, we will investigate which cells are responsible for BAFF production and how γδ T cells influence BAFF production.

Our studies indicated that IL17-γδ T cells can greatly influence not only innate immunity and may have a role in humoral immunity during acute *P. aeruginosa* pulmonary infection. Therefore, to elucidate the effect of IL17-γδ T cells on bacterial clearance and ultimate prognosis, we measured the bacteria load in BALF from mice on day 7 post infection, finding that IL17-γδ T cells help to eliminate bacteria. Considering the role of inducing neutrophil chemotaxis and immunoglobulin production, IL17-γδ T cells help eliminate bacteria through not only innate immunity but also humoral immunity. Given the effect of bacterial clearance, regulation of IL17-γδ T cells improved the survival rate in acute *P. aeruginosa* pulmonary infected mice. Several developmental studies and clinical trials based on active and passive immunotherapy have been performed in the last decades to prevent or treat infections due to drug-resistant bacteria, particularly in immunocompromised patients, (Savoia, 2014), and numerous preclinical trials of γδ T cell immunotherapy have been performed in malignancies (Kang et al., 2009; Bryant et al., 2011; Zhou et al., 2012). γδ T cells might be a suitable immunotherapy target for acute *P. aeruginosa* pulmonary infection. Our findings provide a new understanding of the molecular mechanisms behind the immune function of γδ T cells, which may have implications for γδ T cell-based immunotherapy in *P. aeruginosa* pulmonary infection.

Although we have demonstrated that γδ T cells may influence humoral immunity via IL17, as this study was exploratory and very preliminary, the mechanisms responsible for IL-17-mediated B cell activation and immunoglobulin production still deserve investigation in a dedicated study; future studies will also address how BAFF is involved in this process. These will be the major problems to be addressed in future work.

## Author contributions

TP contributed to the conception, planning and performance of experiments, the interpretation of results and for drafting this manuscript; RT contributed to the conception, planning and performance of experiments and the interpretation of results. Both ML and ZL contributed to the conception of experiments and interpretation of results. Both XW and LT contributed to performance of experiments. JL and HQ designed the study, identified appropriate methods. All authors reviewed and approved this manuscript. TP and RT contributed equally to this work.

### Conflict of interest statement

The authors declare that the research was conducted in the absence of any commercial or financial relationships that could be construed as a potential conflict of interest.

## Abbreviations

BAFF: B cell activating factor of the TNF family
BALF: Bronchoalveolar lavage fluid
CFU: Colony-Forming Units
IL-17: Interleukin 17
IL17-γδT cells: IL17 producing-γδT cells
*P. aeruginosa*: Pseudomonas aeruginosa.
